# Human Amniotic Membrane Dressing as a Non-Surgical Alternative for Extensive Chronic Ulcers: A Comparative Case Study

**DOI:** 10.3390/ijms27114655

**Published:** 2026-05-22

**Authors:** María Ximena Guerbi, Jimena María del Pilar Rodrigo, Matías Fabián Rotela, Rocío Antonella Comito, Esteban Vogel, Enrique Leo Portiansky, Alejandro Berra, Griselda Noemí Moreno, Flavia Mariana Michelini

**Affiliations:** 1 Centro de Medicina Traslacional, Hospital El Cruce (CEMET-HEC), Florencio Varela 1888, Argentina; 2 Comisión de Investigaciones Científicas de la Provincia de Buenos Aires (CIC-PBA), La Plata 1900, Argentina; 3 Instituto de Ciencias de la Salud, Universidad Nacional Arturo Jauretche, Florencio Varela 1888, Argentina; 4 Centro de Educación Médica e Investigaciones Clínicas “Norberto Quirno” (CEMIC), Buenos Aires 1425, Argentina; 5 Servicio de Anatomía Patológica, Hospital El Cruce (HEC), Florencio Varela 1888, Argentina; 6 Laboratorio de Imágenes (CONICET-UNLP), La Plata 1900, Argentina; 7 Banco de Tejidos AMNIOS-BMA, San Martín 1650, Argentina; 8 Consejo Nacional de Investigaciones Científicas y Técnicas (CONICET), Buenos Aires 2290, Argentina; 9 Instituto de Estudios Inmunológicos y Fisiopatológicos (IIFP-CONICET), La Plata 1900, Argentina

**Keywords:** amniotic membrane dressings, wound healing, collagen, immunomodulation, angiogenesis

## Abstract

Chronic wound management remains a significant clinical challenge, requiring adaptive therapeutic approaches to achieve wound closure that nonetheless frequently prove fruitless. Balancing the initial pro-inflammatory response with debris removal and tissue rebuilding remains elusive in most cases, leading to pain, drastic quality-of-life deterioration, and, eventually, amputation. Meanwhile, patient adherence is an overarching theme. Furthermore, non-surgical alternatives that effectively promote tissue rebuilding are essential for patients seeking to avoid further invasive procedures. We report a patient with a recalcitrant ulcer managed using human amniotic membrane dressing (hAM-pe) and a bovine collagen matrix (BCM) in spatially distinct areas as an intra-patient control. Methodology included clinical monitoring and ad hoc molecular and histological analyses to assess inflammatory markers and tissue architecture. Following 59 days of observation, the superior evolution of the hAM-pe-treated zone led to the clinical decision to extend hAM-pe treatment over the adjacent BCM area, resulting in total wound closure. The hAM-pe-treated site demonstrated accelerated closure and clinical resolution of inflammation without the presence of a granulomatous response. Molecular analysis revealed downregulated pro-inflammatory mediators (IL-1β, TNF-α, CXCL-10) and upregulated markers associated with angiogenesis (VEGF, CD34) and tissue repair (Arginase-1). In this case, the non-surgical hAM-pe treatment was associated with a favorable healing trajectory, characterized by superior inflammation resolution and enhanced tissue organization (collagen type I/III maturation). While these descriptive findings suggest the potential advantages of amniotic membrane dressings in promoting advanced tissue repair, they remain limited to this individual observation. Further research in larger cohorts is required to validate these mechanisms.

## 1. Introduction

Skin wounds activate repair mechanisms to restore tissue structure and function [1,2]. However, chronic wounds often stall in a pro-inflammatory M1 macrophage state, failing to transition toward a regenerative M2 phenotype [3]. Current consensus through the TIMERS framework (Tissue, Infection/Inflammation, Moisture, Edge, Repair/Regeneration, Social factors) emphasizes that social factors—including the patient’s will to avoid invasive surgery—are critical to healing outcomes [4]. If a wound fails to heal significantly within four weeks, alternative non-intrusive strategies must be prioritized to reduce unnecessary intrusive practices.

To address the clinical and economic challenges of complex wounds, human amniotic membrane (hAM) offers a bioinductive matrix with antibacterial and anti-inflammatory properties [5,6]. hAM is utilized in ophthalmology to manage corneal perforations and neurotrophic keratopathy by providing an anti-inflammatory, prohealing substrate [7,8]. In oral and maxillofacial surgery, hAM has been used for repair of oral mucosal defects, vestibuloplasty, fistula closure, and management of medication-related osteonecrosis of the jaw, highlighting its versatility as a regenerative biomaterial [9,10]. In vascular surgery, decellularized hAM is fabricated into multilayered tubular grafts that outperform synthetics by promoting endothelialization and maintaining long-term patency [11]. These applications, alongside its use in regenerative medicine as a scaffold for cartilage repair, leverage the membrane’s natural extracellular matrix to facilitate tissue-specific integration [12]. In this study, we employed hAM-pe, a homogenized, lyophilized and gamma-sterilized preparation designed for the gradual release of biological factors and ease of clinical application, which has already been tested in 16 DFU patients without reported adverse effects [13].

This study was motivated by a patient-centered need: an adult male with an extensive gluteal ulcer who declined surgical grafting. Consequently, we describe a comparative evaluation of this non-invasive hAM-pe dressing against a commercial bovine collagen matrix (BCM), analyzing the local histological and molecular responses in a patient seeking to avoid surgical intervention.

## 2. Results

**Figure 1:**
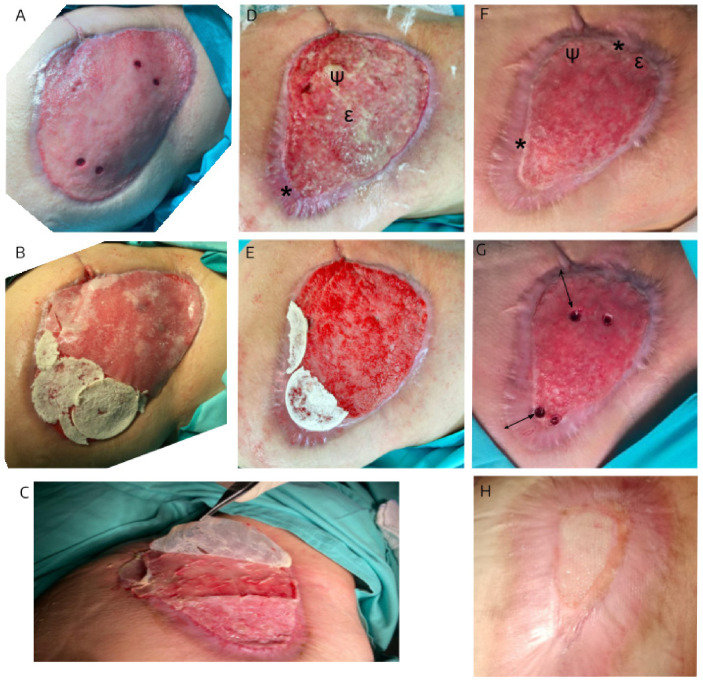
Sequential photographs of the ulcer at various stages of treatment: (**A**) day 0, at the time of biopsy collection, prior to initiating both treatments; (**B**) during the first application of hAM-pe in the lower left area and BCM in the upper right area of the ulcer; (**C**) day 15, after removing the silicone layer covering the BCM; (**D**,**E**) day 39 during one of the hAM-pe reapplications performed every 72 h; (**F**,**G**) day 49, at the time of comparative biopsy collection (double arrows show equal distances from original wound edge) and the initiation of hAM-pe as the sole treatment until discharge (**H**). * indicates visible areas of re-epithelialization. Comparative extent differed between treatment regions at day 49; ψ indicates fibrine depositions and ε indicates edematous regions.

In this individual case, two distinct regions of the same ulcer were treated using different biomaterials: a bovine collagen matrix (BCM) and a lyophilized, homogenized human amniotic membrane dressing (hAM-pe) (Figure 1B). Both treatments were initiated on day 0 in separate wound regions. The BCM dressing was applied once on day 0 and secured with sutures, and after 15 days, the silicone layer was removed (Figure 1C). In contrast, hAM-pe was reapplied every 72 h from day 0 to day 49. The treated areas were followed until day 49. Although both matrices are routinely used for wound bed preparation prior to skin grafting and are expected to promote natural re-epithelialization [14], the hAM-pe-treated area showed a more favorable clinical appearance by day 49, including a broader re-epithelialized margin, less edema, and no visible fibrin-like exudate. Based on the more favorable evolution of the hAM-pe-treated area and the patient’s continued refusal of surgery, hAM-pe treatment was subsequently extended to the remaining wound surface from day 49 onward and continued until complete re-epithelialization was achieved approximately five months after treatment initiation (Figure 1F–H). After 49 days of treatment, under BCM treatment, prominent, irregular granulations were observed, with whitish secretion consistent with fibrin deposits (Figure 1, ψ) and signs of edema (Figure 1, ε). In contrast, the portion treated with hAM-pe exhibited a wider re-epithelialized edge evidenced by a glossy surface (Figure 1, *), suggesting the presence of an incipient keratin layer. Compared with the BCM-treated region, the hAM-pe-treated area showed less visible exudate and edema, which reflects the reduced local inflammatory activity.

Additionally, a histological evaluation was performed on biopsies from the zones treated with BCM and hAM-pe, taken at equivalent distances from the initial wound margin (Figure 1, panel G, double-arrowed lines).

**Figure 2:**
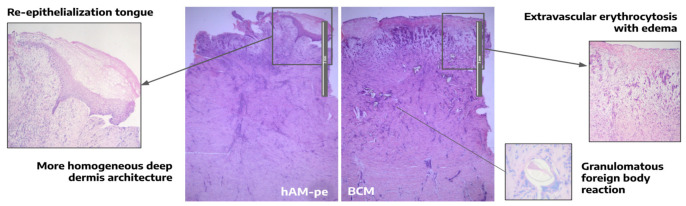
Hematoxylin & Eosin-stained biopsy sections from the hAM-pe and BCM-treated zones, collected on day 49, 2× magnification.

**Figure A1:**
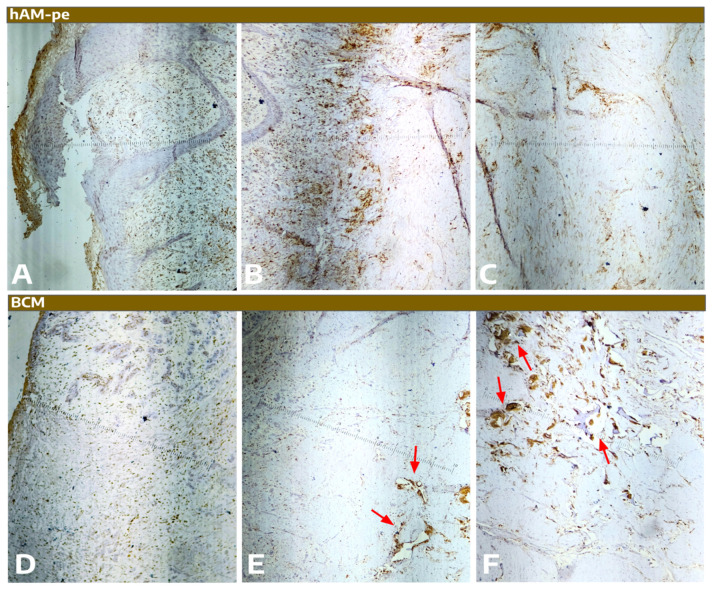
Reported granulomatous reaction supported by CD68 immunohistochemical staining. (**A**–**C**) Sequential overlapping micrographs showing the superficial 3 mm of the hAM-pe-treated skin biopsy. (**D**–**F**) Corresponding images of the BCM-treated area. Red arrows indicate CD68+ histiocytes characteristic of the reported granulomatous reaction. A 1 mm stage micrometer scale is included in the images for reference.

In the zone treated with hAM-pe, a tongue of re-epithelialization was observed, which was not apparent in the biopsy from the BCM-treated zone (Figure 2). These histological findings suggest a more advanced re-epithelialization process following hAM-pe treatment. In addition, a structure composed of immune cells, including epithelioid cells and multinucleated giant cells, characteristic of a foreign-body-type granulomatous reaction was observed and confirmed by CD68 immunohistochemical staining (Appendix A, Figure A1), between 250 and 400 µm in depth, in the zone treated with BCM. This type of structure, which represents a host response aimed at isolating the material and limiting damage to the surrounding tissue, was not observed in the zone treated with hAM-pe. Histological examination revealed that the dermal ECM under BCM treatment appeared disorganized with diffuse unstained white areas indicative of unstructured, edematous connective tissue. In contrast, the area under hAM-pe treatment, both beneath the re-epithelialization tongue and in the surrounding tissue, exhibited homogeneous staining without evidence of extravascular erythrocytosis.

**Figure 3:**
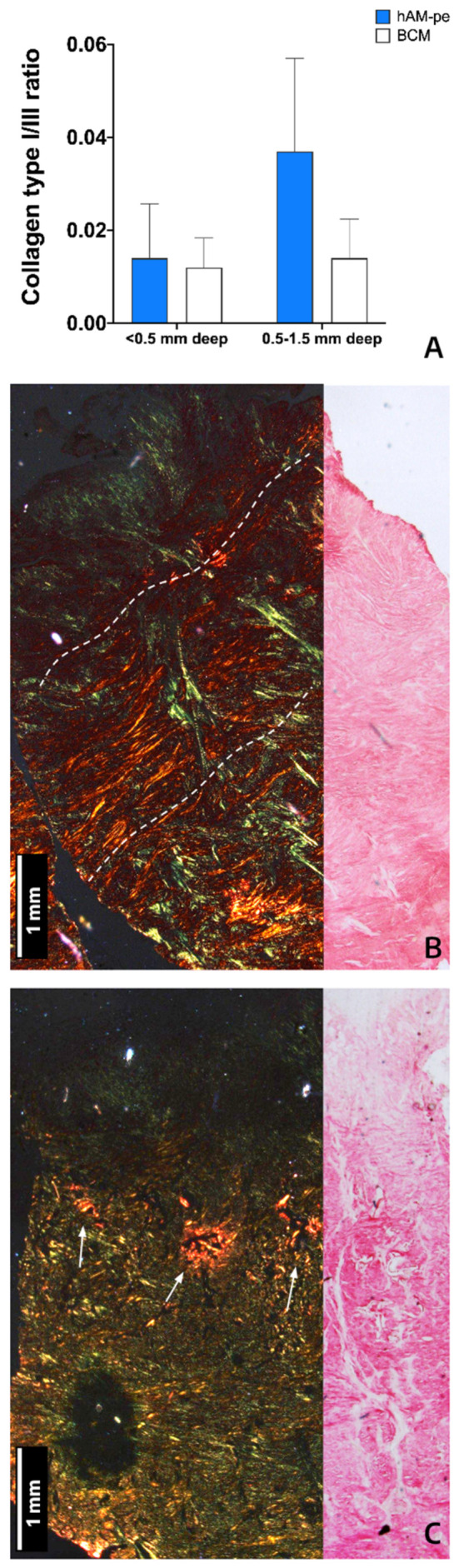
Analysis of collagen deposition and composition after 49 days. (**A**) Bar graph depicting the ratio of type I to type III collagen fibers in newly formed tissue quantified at <0.5 mm deep and 0.5–1.5 mm deep. Quantification was performed using 10 representative regions of interest (ROIs) from each region at 20× magnification. Error bars represent technical replicates SD. (**B**,**C**) Sirius red staining of histological sections from biopsies collected from zones treated with hAM-pe (**B**) and BCM (**C**) after 49 days of treatment. Images on the right show the staining observed under brightfield microscopy and on the left under polarized light. Type III collagen fibers are identified in green, and type I collagen fibers are in red/orange. The dashed line in B highlights the collagen fiber arrangement, while arrows in C indicate collagen deposition. Images taken at 1.25× magnification.

**Figure A2:**
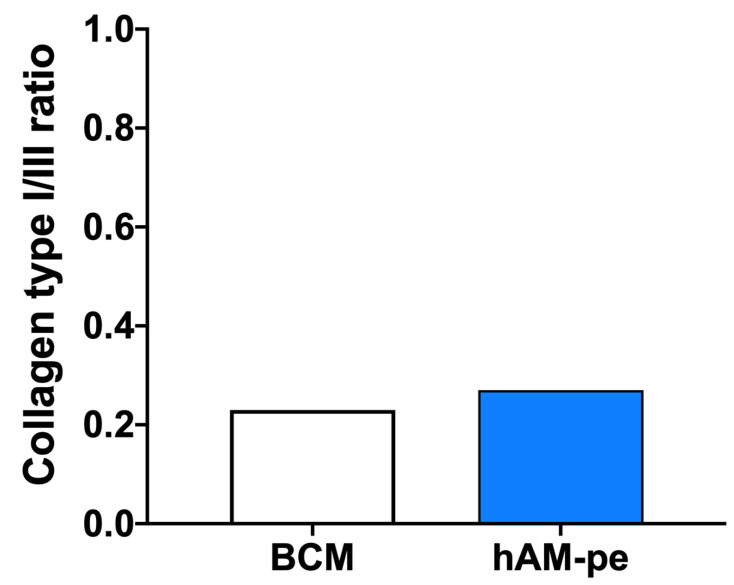
The bar graph represents the ratio of type I to type III collagen fibers, quantified by Picrosirius red technique, in a complete 20× magnification scan of the regions on top of which treatments were applied. No statistical comparison was performed.

To assess the composition and spatial arrangement of collagen fibers, biopsy samples were stained with Picrosirius red (Figure 3). The ratio between type I and type III collagen fibers provides insight into whether the tissue has a normal composition or presents alterations [15,16]. Day 0 biopsies confirmed that no initial differences existed between the regions assigned to each treatment (Appendix B, Figure A2). Assuming that the healing process occurs from the deepest layers of the ulcer toward the surface, the most recently synthesized tissue would be that closest to the surface, where treatments were applied. No differences were observed in the superficial provisional matrix: equal collagen type I/III ratios under both treatments, with an abundance of type III collagen, a hallmark of granulation tissue itself, were observed. However, at a depth of 0.5–1.5 mm, the hAM-pe-treated zone exhibited a higher collagen I/III ratio, indicative of ECM maturation. Accordingly, in the newly synthesized tissue layer over the ulcer bed where BCM was applied, the collagen deposition appeared focal and irregularly organized, particularly in areas suggestive of a foreign-body-type granulomatous reaction at a depth of 1500 to 3000 µm (Figure 3, panel C, arrows). In contrast, hAM-pe dressings were associated with a more fibrillar-like collagen organization, appearing as parallel red/yellow lines in Figure 3, panel B (dashed line). Finally, the comparison between the deepest regions of each biopsy holding the tissue beneath the original bed ulcer is useful to evaluate pre-existing-tissue maturation under the effects of each treatment. Whole-section scanning of this region also showed a higher collagen I/III ratio under hAM-pe treatment, suggesting an advanced progression of a remodeling process.

Given that many of the aforementioned estimates rely on Picrosirius red staining to differentiate collagen fiber types, and acknowledging the reported limitations of this method [17], we further investigated these hypotheses by analyzing molecular marker expression changes. The results of these analyses are presented in the following sections.

**Figure 4:**
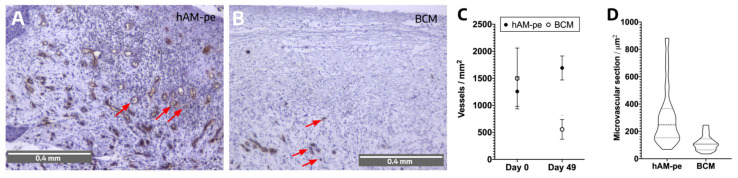
CD34 immunohistochemical staining in treated skin biopsies. Representative images at 10× magnification of sections under hAM-pe (**A**) and BCM (**B**) treatments at day 49, showing three structures fulfilling the criteria for vascular identification, marked with red arrows. (**C**) Comparison of microvessel density between treatments at both timepoints at 2× magnification. (**D**) Comparison of microvascular cross-sectional area in both zones at the end of each treatment at 10× magnification. Error bars represent technical replicates SD.

**Figure A3:**
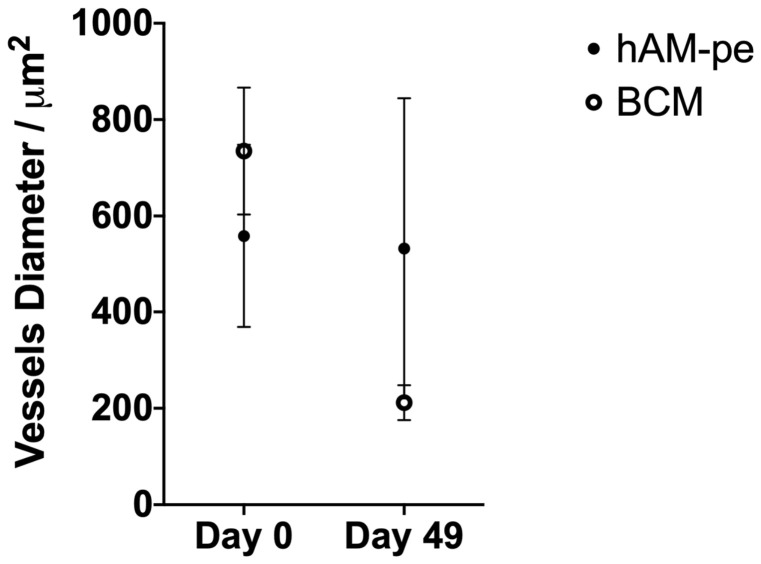
Comparison of microvascular cross-sectional area in both zones, in the superficial region, at the beginning and the end of each treatment at 10× magnification. Error bars represent technical replicates SD. Mean +/− SD of technical replicates.

In order to analyze the vascularization state of the new tissue in the wound under both treatments, the cell surface marker CD34 was analyzed on biopsy sections. CD34 is a robust and functional marker of vascularization because its detection reflects the presence and activity of key cells involved in vascular formation and remodeling [18]. No differences were identified in the number of vessels present in the superficial region of the two initial biopsies (Figure 4C, day 0), nor were differences observed in vessel caliber (Appendix C, Figure A3). Both observations support an equivalent vascularization between the areas set to receive the two treatments. After 49 days of treatment, images were significantly different under each treatment (Figure 4A,B). The zone treated with hAM-pe exhibited an increased count of CD34-positive circular structures indicative of blood vessels (Figure 4C, day 49). Additionally, a larger vessel diameter was observed in the same zone, as revealed by the microvascular sections of all detected structures examined at higher magnification (Figure 4D). These findings indicate that hAM-pe treatment significantly enhanced both the number and caliber of newly formed blood vessels over time compared to BCM treatment. When these processes are impaired, they can lead to edema and inadequate tissue oxygenation, ultimately compromising the healing process [19].

**Figure 5:**
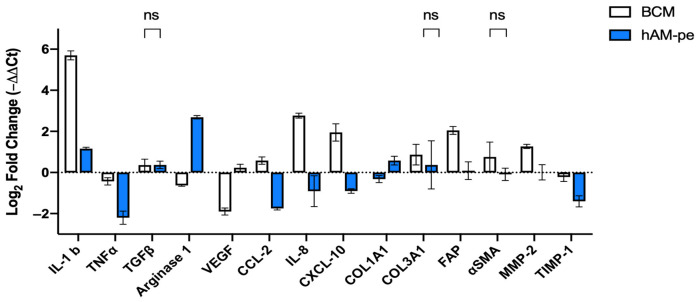
Relative expression of biomarkers in dermal ulcer tissue. Light blue bars represent hAM-pe treatment and white bars represent BCM treatment. Data are expressed as mean Log2 Fold Change (−ΔΔCt) +/− SD of technical replicates. All differences between treatments exceed the 3σΔ threshold except where indicated (ns).

The clinical and histological observations described above suggested distinct local tissue responses under the two treatment conditions. To further characterize these findings, the expression of selected molecular markers was also analyzed, as described in Section 4 and represented in Figure 5 and Table 1.

Regarding inflammation markers, hAM-pe treatment resulted in lower expression of TNF-α (ΔLFC = −1.77) and IL-1β (ΔLFC = −4.54) compared to BCM. Conversely, Arginase 1 expression was higher under hAM-pe (ΔLFC= 3.33), while no significant differences were observed for TGF-β. This profile suggests a modulation toward a regenerative state under hAM-pe [3,20,21]. Chemotaxis and vascularization markers also exhibited differential expression. Under hAM-pe, VEGF expression was higher (ΔLFC = 2.13) [22,23], whereas chemotactic markers CXCL-10, IL-8, and CCL-2 showed lower expression levels relative to BCM (ΔLFC < −2.30). These findings indicate reduced recruitment of inflammatory cells and sustained angiogenic signaling in the hAM-pe-treated area [24]. Regarding ECM deposition and remodeling, hAM-pe showed higher COL1A1 expression (ΔLFC = 0.90), while no significant differences were detected for COL3A1 or α-SMA. Enzymatic markers associated with matrix turnover, including Gelatinase MMP-2 (ΔLFC = −1.26), protease inhibitor TIMP-1 (ΔLFC = −1.18), and serine-protease FAP (ΔLFC = −1.95) [25,26], were lower under hAM-pe compared to BCM treatment. While MMP-1 was initially undetectable, its induction was observed in both treatments by day 49, being 10-fold higher in the hAM-pe-treated zone. These findings suggest that in the hAM-pe-treated area, the replacement of type III collagen with a more mature type I collagen matrix is already underway, whereas the BCM zone remains in an earlier stage of enzymatic remodeling [27].

## 3. Discussion

In this single-case intra-patient comparative study, hAM-pe was associated with the resolution of persistent inflammation and promotion of angiogenic features, as reflected by M2-like profiling and increased vascularization (VEGF/CD34+) [22,28]. In contrast, the BCM-treated area showed persistent pro-inflammatory features and foreign-body reactions, while the hAM-pe-treated region exhibited findings consistent with more advanced remodeling, including a higher collagen I/III ratio [29,30,31,32,33,34,35].

Unlike conventional dermal substitutes that often require subsequent split-thickness skin grafting, the hAM-pe treatment used for this patient was associated with a non-surgical healing trajectory, facilitating spontaneous epithelialization from wound edges and adnexal structures. By avoiding the need for sutures or operating room intervention, this approach aligned with the patient’s preference to avoid surgery and was accompanied by a successful home-based recovery with improved quality of life.

Although these findings derive from a single-case report and cannot be generalized to the broader population, they suggest the potential of hAM-pe as a regenerative alternative deserving of further investigation. Larger controlled studies are required to determine its efficacy, safety, and reproducibility in complex wound management.

## 4. Materials and Methods

### 4.1. Patient and Skin Samples

This study was approved by the Ethics Committee of Research of the Hospital El Cruce, Argentina. The patient agreed to participate in this study, as well as to have its results published, and written informed consent was obtained from him. This study was developed in compliance with the regulations concerning current legal aspects in the Argentine Republic, adhered to requirements regarding respect for patients’ rights, and did not violate any national or international ethical guidelines, as reflected in the approval by the institution’s Human Research Review Committee.

An adult male patient with a 60 cm^2^ gluteal ulcer (post-injection fasciitis, 1-month VAC) was referred while undergoing chemotherapy for multiple myeloma. To defer surgery per patient request, a comparative treatment was applied to two ulcer regions: commercial BCM vs. hAM-pe. The BCM was sutured and its silicone layer removed on day 15. while hAM-pe was applied every 72 h. For histological and molecular analysis, 0.5 cm punch biopsies were collected on days 0 and 49. Clinical progress was documented via photography.

### 4.2. Treatments

The BCM employed is a commercial product described as a three-dimensional porous bi-layer sterile matrix consisting of type I, purified, stabilized, freeze-dried bovine collagen and a polyester-reinforced silicone layer acting as a pseudo-epidermis [36].

hAM-pe dressingsare obtained by homogenizing, lyophilizing and sterilizing human amniotic membranes for which extensive donor screening is performed to ensure donor suitability. Material is obtained from the placenta after cesarean delivery, following informed consent. Together with placentas, a blood sample from the mother is provided and assessed for HIV, HBV, HCV, CMV, HTLV, *Treponema pallidum*, *Brucella* spp., Trypanosoma cruzi, and Toxoplasma gondii. Placentas are stored at 4 °C in sterile saline and transported to the AMNIOS BMA Tissue Bank, San Martín, Argentina. Primary processing is done within 24 h of the cesarean and involves manually separating the AM from the placenta under a class 100 biological safety cabinet. The hAM is homogenized with a handheld homogenizer and transferred to circular molds of 5.5 and 6.0 cm diameter for the lyophilization process using a BK-FD 10P lyophilizer. Then, the hAM-pe is packed inside 2 bags under a class 100 biological safety cabinet. After that, hAM-pe is sterilized through gamma radiation. The dressings are provided in circular shapes of 5.5 and 6.0 cm in diameter and 1.2 ± 0.1 mm in thickness. All processes are carried out following Good Manufacturing Practices approved by the National Administration of Drugs, Foods and Medical Technology (ANMAT, Ciudad Autónoma de Buenos Aires, Argentina) and AMNIOS BMA Tissue Bank, approved by the National Institute for Ablation and Implant (INCUCAI, Ciudad Autónoma de Buenos Aires, Argentina). Furthermore, control samples are sent to the microbiology service to determine the bioburden and to ensure sterilization has been achieved with the range of gamma radiation employed (15–25 kGy). hAM-pe remains in quarantine until all controls are completed. If stored at room temperature and away from direct light, hAM-pe has a shelf life of 3 years [13].

### 4.3. Histological Analysis

Four FFPE skin biopsies (5 µm) were blindly analyzed using H&E and Picrosirius red staining [37]. Polarized microscopy and ImageJ (Version: 2.14.0/1.54f) thresholding quantified collagen I/III ratios. IHC for CD34 (1:50) and CD68 (1:100) utilized HRP/DAB detection. Vessel density and diameter within the superficial 200 µm were quantified via the “Hot Spot” morphometric technique on CD34+ structures across selected ROIs [38].

### 4.4. Molecular Biology Analysis

Four biopsies were homogenized for RNA extraction (Tiangen, Beijing, China) and triplicate reverse transcription (iScript, Bio-Rad, Hercules, CA, USA). Relative gene expression (Actin-normalized) was analyzed via qPCR (iQ Bio-Rad, CFX96) using a 40-cycle protocol and melting curve verification. Primer sequences are shown in Table 2. Relative mRNA expression was calculated via the −ΔΔCt method which provides a symmetric Log2 scale for up- and downregulation. Given N = 1, significance was defined by technical robustness: effects were significant if they exceeded three times the technical standard deviation, ensuring a 99% confidence interval against experimental noise.

## 5. Conclusions

In this single-patient case, treatment with hAM-pe was associated with more favorable clinical, histological, and molecular features compared with BCM, including resolution of local inflammation and findings consistent with more advanced tissue remodeling. The observed non-surgical healing trajectory and good tolerability highlight the potential of hAM-pe as a supportive approach in complex wound management. However, these findings are limited to intra-patient observation and cannot be generalized. Further controlled studies are required to evaluate the efficacy, safety, and reproducibility of this strategy.

## Figures and Tables

**Table 1:** Differential expression analysis (ΔLFC) and statistically significance thresholds (3σΔ) for dermal ulcer biomarkers. Double asterisks ** denote statistical significance exceeding the 3σΔ threshold, corresponding to a 99.73% confidence level, which aligns with the standard *p* < 0.01 classification; ns stands for no significance.

Biomarker	ΔLFC (hAM-pe − BCM)	Threshold (3σΔ)	Significance
IL-1β	−4.54	0.70	**
TNF-α	−1.77	1.09	**
TGF-β	0.01	1.02	ns
Arginase 1	3.33	0.27	**
VEGF	2.13	0.71	**
CCL-2	−2.33	0.57	**
IL-8	−3.68	2.29	**
CXCL-10	−2.85	1.31	**
COL1A1	0.90	0.81	**
COL3A1	−0.50	3.80	ns
FAP	−1.95	1.42	**
α-SMA	−0.22	2.34	ns
MMP-2	−1.26	1.16	**
TIMP-1	−1.18	1.04	**

**Table 2:** Primer sequences used for qPCR analysis.

Amplicon	Forward Primer	Reverse Primer
β-actin	CCT GGC ACC CAG CAC AAT	GCC GAT CCA CAC GGA GTA CT
IL-1β	TAC GAA TCT CCG ACC ACC ACT ACA G	TGG AGG TGG AGA GCT TTC AGT TCA TAT G
TNF-α	AAC CTC CTC TCT GCC ATC AA	CCA AAG TAG ACC TGC CCA GA
TGF-β	ACC CAC AAC GAA ATC TAT GAC	GCT CCA CTT TTA ACT TGA GCC
Arginase 1	GTT TCT CAA GCA GAC CAG CC	GCT CAA GTG CAG CAA AGA GA
VEGF	CAC TGC CTG GAA GAT TCA	TGG TTT CAA TGG TGT GAG GA
CCL-2	CGC CTC CAG CAT GAA AGT CT	ATG AAG GTG GCT GCT ATG AGC
IL-8	CAC CGG AAG GAA CCA TCT CA	GGA AGG CTG CCA AGA GAG C
CXCL-10	TCC ACG TGT TCA GAT CAT TGC	TGA TGG CCT TCG ATT CTG G
COL1A1	CGA AGA CAT CCC ACC AAT CAC	TCA TCG CAC AAC ACC TTG C
COL3A1	CTG GTC CTG TTG GTC CAT CT	ACC TTT GTC ACC TCG TGG AC
FAP	ATG AGC TTC CTC GTC CAA TTC A	AGA CCA CCA GAG AGC ATA TTT TG
α-SMA	AGG GAG TAA TGG TTG GAA TGG	TGA TGA TGC CGT GTT CTA TCG
MMP-1	TCG CTG GGA GCA AAC ACA	TTG GCA AAT CTG GCG TGA A
MMP-2	CCT CTC CAC TGC CTT CGA TA	GCC TGG GAG GAG TAC AGT CA
TIMP-1	CGC TGA CAT CCG GTT CGT	GTG GAA GTA TCC GCA GAC ACT CT

## Data Availability

The data presented in this study are available in the article. Further inquiries can be directed to the corresponding author.

## Abbreviations

The following abbreviations are used in this manuscript:

α-SMA	Alpha-Smooth Muscle Actin
BCM	Bovine Collagen Matrix
CCL-2	Motif Chemokine Ligand 2 (Monocyte Chemoattractant Protein-1, MCP-1)
CD34	Cluster of Differentiation 34 (marker of hematopoietic and endothelial progenitor cells)
COL1A1	Collagen Type I Alpha 1 Chain
COL3A1	Collagen Type III Alpha 1 Chain
Ct	Threshold Cycles
CXCL-10	C-X-C Motif Chemokine Ligand 10 (Interferon gamma-induced protein 10)
DFU	Diabetic Foot Ulcer
ECM	Extracellular Matrix
FAP	Fibroblast Activation Protein
hAM	Human Amniotic Membrane
hAM-pe	Lyophilized Homogenized Human Amniotic Membrane Dressings Sterilized by Gamma Radiation
HIV	Human Immunodeficiency Virus
IL-1β	Interleukin 1 Beta
IL-8	Interleukin 8
MMP-1	Matrix Metalloproteinase-1
MMP-2	Matrix Metalloproteinase-2
RNA	Ribonucleic Acid
ROI	Region of Interest
qPCR	Quantitative Polymerase Chain Reaction
TGF-β	Transforming Growth Factor Beta
TIMP-1	Tissue Inhibitor of Metalloproteinases-1
TNF-α	Tumor Necrosis Factor Alpha
VAC	Vacuum-Assisted Closure
VEGF	Vascular Endothelial Growth Factor
